# Genetic polymorphism of *miRNA-196a* and its target gene *annexin-A1* expression based on ethnicity in Pakistani female breast cancer patients

**DOI:** 10.12669/pjms.35.6.1322

**Published:** 2019

**Authors:** Amena Rahim, Muhammad Afzal, Abdul Khaliq Naveed

**Affiliations:** 1 Prof. Amena Rahim, Department of Biochemistry, Islamic International Medical College, Riphah International University, Islamabad, Pakistan; 2 Dr. Muhammad Afzal, Research Officer, Department of Biochemistry, Islamic International Medical College, Riphah International University, Islamabad, Pakistan; 3 Prof. Abdul Khaliq Naveed Department of Biochemistry, CMH Lahore Medical College, National University of Medical Sciences, Islamabad, Pakistan

**Keywords:** Single nucleotide polymorphism, Breast cancer, miR-196a, annexin-A1, ANXA1 gene expression

## Abstract

**Objective:**

To evaluate the association of *miR-196a* rs11614913 C/T genetic variation and its target gene *annexin A*1 mRNA expression with breast cancer risk in Pakistani female ethnicities.

**Methods:**

This case control study, conducted from March 2017 to November 2018 included 295 breast cancer patients, 295 controls of three Pakistani ethnicities and archived 100 samples of cohort group for genotyping and expression profiling. Genotyping of *miR-196a* (rs11614913 C/T) was done by ARMS PCR technique. Annexin-A1 (*ANXA1)* mRNA expression was measured with qRT-PCR and detection of protein expression of ANXA1 was done by immunohistochemistry.

**Results:**

CC homozygous genotype of *miR-196a* rs11614913 was present in 81.4% of cases and 73.9% controls. C/T polymorphism was found to be significantly associated with decrease risk of breast cancer (OR=0.25 (0.11- 0.58, *p* <0.05). Similar trend was seen with the minor T allele (OR=0.55 (0.39-0.77, *p* <0.05, and both dominant and recessive models (OR=0.64; *p*=0.02 and OR=0.26, *p*=0.00). In the KPK ethnic group significant decrease association with breast cancer risk was observed (OR= 0.22 (0.09-0.53, *p* < 0.05). Immunohistochemical staining showed loss of ANXA1 protein expression in 72 samples, and significant association was observed with pathological type *p*=0. 00 and triple negative receptor status *p*=0.03 and with genotypes of *miR-196a* *p*=0.00. Increase relative expression of 2.81± .88 by qPCR analysis of *ANXA1* mRNA was noted with TT genotype.

**Conclusions:**

Our results demonstrate that *miR-196a* rs11614913 C/T polymorphism is associated with a decreased risk and loss of protein expression in breast cancer in the Pakistani population.

## INTRODUCTION

Breast carcinoma is the most common cancer among women. There are 611,000 breast cancer (BC) deaths each year and it was the 18^th^ leading cause of disability-adjusted life-years in 2017.^1^ Pakistan is a developing country with low health care investments. In HAQ index, Pakistan is ranked 154^th^ out of 194 countries in the world.^2^ MicroRNAs are non-coding 20-22 nucleotide long single stranded RNA molecules. They base pair to the 3` untranslated region (3`UTR) of target mRNA to regulate gene expression. Most miRNAs are down regulated in cancer cells and this repression causes cellular transformation which can lead to tumor development and progression*miR-196a* (rs11614913) C to T nucleotide substitution in the 3`p end of the mature strand sequence can modify its expression and function and this can lead to increased cancer susceptibility.^3^ *miR-196a* target genes are involved in cell cycle, differentiation and apoptosis. Luthra et al., reported *annexin-A1* *(ANXA1*) as one of the target genes of *miR-196a*.^4^ *ANXA1* regulates physiological mechanisms such as hormone secretion, apoptosis, exocytosis and signal transduction. *miR-196a* acts by targeting *ANXA1* and suppressing it, thereby promoting cell proliferation and suppressing apoptosis and this provides evidence for its oncogenic potential.^5^ Expression levels of *ANXA1* detected in different cancers are not consistent. In certain tumors such as head and neck, esophageal squamous cell and prostate cancers they are down regulated and in others like glioma and oropharngeal cancers they are up regulated.^6^

Pakistan is a multiethnic state with five provinces and different ethnicities. This study was aimed to assess the risk association of *miR-196a* rs11614913 C/T variant and the relationship of *miR-196a* genotype with expression of its target gene *ANXA1* in BC cases. Once we get an insight into the pattern of these particular miRNAs, polymorphism in our population it will help the oncologist in detection of breast cancer in its very early stage as individuals with high risk genetic variants of these miRNAs can be screened periodically to avoid un detection or late detection of the breast cancer and they will be able to routinely check for any symptoms without undergoing any invasive tests. According to our information it is the first study to study this association in our population.

## METHODS

A case control study with a non-probability convenient sampling technique was conducted from March 2017 to November 2018. A total of 295 patients from two hospitals - Holy family hospital, Rawalpindi and NORI hospital, Islamabad were included. The Ethical Review Committee of Islamic international Medical College approved the study (Appl.#Riphah/IRC/18/0356), and informed written consent was taken from the study subjects. Patients were recruited for the study without any restriction on age or disease histology and included three major ethnic groups of Pakistani females including Punjabi, Khyber Pakhtunkhwa and Kashmiri females. Their demographic and clinical data was collected from the hospital files. They included diagnosed cases, both with family history or sporadic cases of breast cancer; all age groups with different stages of breast tumors. Breast cancer patients and healthy controls were not related but belonged to same ethnic groups. Controls were recruited from within the general population and were frequency matched to cancer patient for age, gender and ethnicity. Their inclusion criteria was; absence of prior history of cancer or any precancerous condition, absence of any chronic disease and females with any first degree relative with breast cancer. Those having any other accompanying malignancy both presently and in the past were excluded. *Annexin A1* gene expression was studied from 100 FFPE (Formalin –Fixed, paraffin embedded) tissue blocks of clinically diagnosed BC cases with different stages and ages.

Genomic DNA from blood was extracted using 5-7% chelex (Bio-Rad). The DNA concentration was measured at 260/280 nm (Nano drop 2000c, Thermo Fischer Scientific). The genotyping of *miR-196a* (rs11614913) C/T gene variation was detected by allele specific T-ARMS-PCR technique.^7^ Four primer set, two outer; forward outer used for the amplification of C and T alleles. Common Primer -R5`-GGCATAAAGCAGGGTTCTCCAGACTTGT-3` and Common Primer F5`GGTCCCATTTCACCAGATTTTTCCTGAG3` and two inner allele specific; Inner forward C-allele (5`AGTTTTGAACTCGGCAACAAGAAAGTGC-3`) and reverse T-allele 5`- CGACGAAAACCGACTGATGTAACTGAGA -3` were used for the amplification of C and G alleles. PCR reactions were performed in a thermal cycler (Major Sciences, USA), in a total volume of 20μl containing 1.5μl (approximately 100 ng/μl) of DNA template, 4μl master mix (12.5mM MgCl_2_) (5X FIREPol ^(R)^ Master Mix- Solis BioDyne), 1μl primer mix (each 10 *p*mol/μl) and PCR grade water up to 14.5μl. PCR cycling conditions were optimized as 1 cycle for holding temperature at 95°C for 5 mins; 30 cycles at 95°C for 30 secs, 59°C for 25 secs, 72°C for 30 secs and final extension at 72°C for 7 minutes. Separation of amplified products was done on 1.5% agarose gel which consisted 15μl ethidium bromide solution of 0.5 μg /μl per 100ml agarose solution. The resolved DNA bands were documented in gel documentation system (G-Box, syngene USA). The PCR amplicons sizes of *miR-196a* (rs11614913) C/T were as follows: C- allele153 bp, T-allele 199 bp and 297 bp for internal control bands. Internal control primers were used for the common amplification of DNA sequences in genotyping. About 10% of samples were re-assayed by using primer sequences from two different companies. For the separation of DNA from FFPE tissue samples, a Nucleo Spin kit (NucleoSpin® DNA FFPE XS (MACHEREY- NAGEL) was used. The NucleoSpin kit (NucleoSpin® RNA FFPE XS (MACHEREY- NAGEL)) was used for the separation of RNA from FFPE samples following manufacturer’s instructions. For RNA reverse transcription to cDNA, the FIREScript RT cDNA Synthesis KIT (Solis BioDyne) cat No; 08-24-00001 was used. cDNA synthesized was used for the measurement of *annexin-A1* gene expression and after quantification by SYBR green assay (MiniOpticon real-time PCR detection system with CFX Manager™ software- Bio Rad). mRNA expression levels were normalized to that of β-actin.

Mouse/Rabbit Immuno-Detector DAB HRP Brown Detection System (Bio SB, USA) was utilized for immunodetection. Antibodies used were Annexin-A1 (MRQ 3) and Mouse Monoclonal Antibody (CELL MARQUE- SIGMA- ALDRICH.) (cat No; 221M-17). Immunohistochemical staining for annexin A1 was carried out on 3-4 µm FFPE embedded breast tissues according to standard protocol. A histopathologist analyzed ANXA1 tissue section staining intensity.

### Statistical Analysis

Descriptive statistics for frequency distributions, χ^2^ test for the association of genotypes with clinicopathological features, allele frequency difference between cases and controls and ANXA1 protein expression. Odds ratio (OR) and 95% confidence interval (CI) were calculated for the association of genotype, breast cancer risk and ethnicity in cases and controls. Univariate and multivariate logistic regression models were applied. *p- value* < 0.05 was taken as significant. Data analysis was done by SPSS version 22.0 (SPSS, Inc; IL, USA).

## RESULTS

### Descriptive statistics

The study group consisted of 295 breast cancer cases (median age 56.2±10.06 years) and 295 controls (median age 56.0 ± 10.2 years), sporadic cases (88.1%) and familial cases (11.9%). The left side involvement was 59%, tumor size 2-5 cm (73.9%), histological type infiltrating ductal carcinoma (79.3%), and grade II and stage II (82% and 47.1%) respectively.

### The genotype frequency of miR-196a compared with different clinicopathological features

Age, size, pathological type, grade and stage of the disease showed a significant association (*p*=0.00). Family history, side and receptor status did not show any association.

### Genotypic frequency between cases and controls

In dominant model, the minor allele T showed a decreased association with breast cancer risk (OR=0.64, 95%CI=0.43-0.95, *p=*0.02*)* and a similar association existed in the recessive model (OR=0.26, 95% CI=0.11-0.59, *p*= 0.00) and in the allelic model (OR=0.55, 95%CI=0.39-0.77, *p*=0.00; Table-I).

**Table I:** Association of *miR-196a* genotype and allelic frequencies in cases and Controls.

SNP 196 C>T	Cases N=295	Controls N=295	OR (95% CI)	p - Value

Genotype	n(%)	n(%)		
CC	240 (81.4)	218 (73.9)	Ref 1	
CT	47 (15.9)	49 (16.6)	0.87 (0.56-1.35)	0.53
TT	8 (2.7)	28 (9.5)	0.25 (0.11-0.58)	0.00

*Dominant Model*				

CC	240 (81.4)	218 (73.9)	--	
TT+CT	55 (18.6)	77 (26.1)	0.64 (0.43-0.95)	0.02

*Recessive Model*				

CC+CT	287 (97.2)	267 (90.5)	--	
TT	8 (2.7)	28 (9.5)	0.26 (0.11-0.59)	0.00

*Alleles*				

C	527 (89.3)	485 (82.2)	--	
T	63 (10.6)	105 (17.7)	0.55 (0.39-0.77)	0.00

OR – Odds Ratio, CI- confidence Interval, P <0.05 – statistically significant.

A significant association between *miR-196a* genotype and breast cancer risk was observed only in the Khyber Pukhtunkhwa (KPK) ethnic group (OR= 0.22, 95%CI= 0.09-0.53, *p*=0.00). No association was observed in the other two ethnic groups i.e. Punjabi and Kashmiri, Table-II.

**Table II:** Association of *miR-196a* genotype with ethnicity in cases and control groups.

Ethnicity/Genotype	Cases N=295 n(%)	Controls N=295 n(%)	OR(95%CI)	p - Value
Punjabi	n=195 (%)	n=198 (%)	CC vs TT+CT	
CC	156 (80)	159 (80.3)	1.01 (0.62-1.67)	0.92
CT+TT	39 (20)	39 (19.6)		
Kashmiri	n=41(%)	n=39(%)		
CC	34 (82.9)	27 (69.2)	0.46 (0.16-1.33)	0.15
CT+TT	7 (17)	12 (30.7)		
KPK*	n=59 (%)	n=58(%)		
CC	50 (84.7)	32 (55.2)	0.22 (0.09-0.53)	0.00
CT+TT	9 (15.2)	26 (44.8)		

### Univariate and multivariate analysis

The patients with genotypes CC (OR=4.39, 95%CI=1.97-9.79, *p<*0.001) and CT (OR=3.55, 95%CI=1.48-8.52, *p=*0.004) were found to be more likely to develop breast cancer compared to the TT genotype.

At a univariate level the estrogen receptor (ER), progesterone receptor (PR) and HER2 receptor status were found to be statistically significantly associated with all three genotypes CC, CT and TT (*p<*0.001), while the combined parameters of ER+PR+HER2 showed no association at the univariate level (*p=*0.57).

Multinomial logistic model for association with *miR-196a* genotype, PR was found to have a protective response and was less likely to be associated with the CT genotype (OR=0.21, 95%CI=0.05-0.89, *p=*0.03).

Out of 100 FFPE tissue samples loss of expression was seen in 72% cases and positive expression was seen only in 28% cases. ANXA1 expression showed a highly significant association with pathological type *(p*=0.00), and triple negative receptor status (*p*=0.03). However no significant association was seen with age, stage, size and grade.

**Fig. 1:**
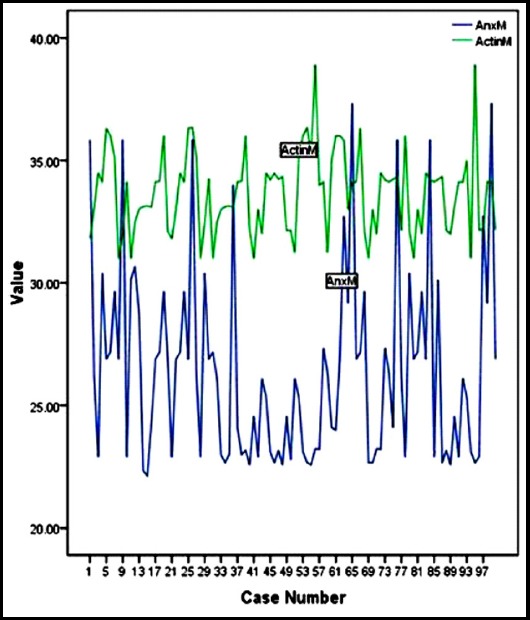
Real time PCR analysis of *annexinA1* expression in breast cancer samples in different genotypes of *miR-196a*.

**Fig. 2:**
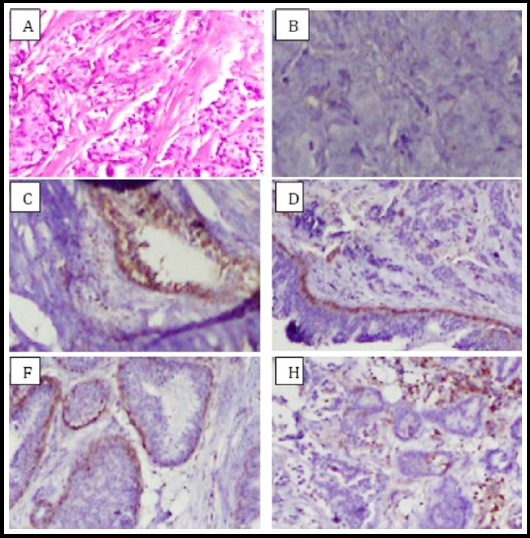
Representive pictures of Annexin A1 (ANXA1) expression in breast tissues. (A) Hematoxylin and eosin stained primary breast cancer tissue. Immunohistochemical stained breast cancer samples for ANXA1 marker (B and C) negative and positive sample built in control. (D) Positive in myoepithelium cells and slight positive in tumor part. (F and H) ANXA1 expression positive in ductal carcinoma In-situ and primary breast cancer case. 200x magnification.

About 28% were positive for ANXA1 expression and 72% showed negative expression (*p*=0.00). Negative expression of the genotypes CC, CT and TT was 62.5%, 34.7% and 2.8%, respectively. After normalization with reference to the *β-actin* gene, mean levels of 33.68 ± 1.68 for *β-actin* gene and 26.40 ± 3.88 for *ANXA1* were seen. Fig. 1. Immunohistochemical stained breast cancer samples for ANXA1 are shown in Fig. 2.

## DISCUSSION

Worldwide incidence of cancer is increasing yearly and the responsible factors are mostly unknown. Genetic factors have been implicated as one of the major cause of cancer, and over the past few decades’ microRNAs have established their role in the manifestation of various cancers including breast cancer. MiRNAs are involved in the normal physiological mechanisms, which are regulated by targeting their concerned genes involved in the regulation of various processes like cellular proliferation and apoptosis. Deregulation like single nucleotide polymorphisms may predispose them towards malignant alterations.^8^ *miR-196a* have been implicated by several studies in different cancers, In vertebrates the gene family for *miR-196a* is located on chromosome 12 in the region of homeobox clusters which encode homeo domain containing transcription factors. Hox proteins act as anti-apoptotic in BC cells by interfering with p53 genes. miR-196a rs11614913 variant may intensify or diminish the translation of its protein.^9^ According to Alshatwi *et al* the expression levels of *miR-196a* (rs11614913) are very high in both pancreatic and breast cancers as compared to normal tissues and this increase expression is associated with decrease survival rates in carcinoma pancreas, and increase chance of developing breast cancer.^10^ *miR-196a* variant not only affects the levels of mature *miR-196a* but also influences the expression levels of its target gene.^11^ Different studies are conducted to study a link between *miR-196a* (rs11614913) and breast cancer risk. Some studies report it as a protective factor while others like Omrani *et al* and Qi P *et al* observed increase susceptibility to breast cancer.^12^,^13^ This current study was conducted to study the risk association between *miR-196a* C/T rs11614913 polymorphism and breast cancer in Pakistani population and its different ethnicities. To date studies done so far have tried to find out the association between this SNP in various populations like Chinese, Caucasians, Italians and Germans etc.^14^-^16^ In the subgroup analysis the homozygous CC genotype had the highest frequency in all the ethnicities of the Pakistani population in both cases and controls. The frequency of the C allele was also found to be greater in both the cases and the controls as compared to the T allele. C was the major allele in both Brazilian and Iranian populations.^17^,^18^ Our study confirmed decreased susceptibility to breast cancer with the TT genotype. Hu *et al* indicated that the TT genotype was protective compared to the CC/TT genotype.^19^ When the risk association was analyzed with disease free survival, the C-allele of *miR-196a* exhibited worse prognosis than the others.^3^ Similarly a meta-analysis of 15 studies also correlated CC genotype with increased risk of breast cancer in comparison to the TT genotype.^20^ When analyzed by ethnicity, it was observed that there was a significant association with BC only in the population with KPK ethnicity. It means that SNPs show diversity among and within the ethnic groups of a population. Meta-analysis observed decreased risk of BC in both allelic and recessive models,^21^ and another analysis indicated an increased association with cancer susceptibility of the homozygous CC genotype among Asians and a decreased risk among Caucasians.^14^ No such relationship was observed in Australian,^15^ German and Italian women.^16^ In contrast Hu *et al*.^19^ established an increased association and Hoffman *et al*.^11^ evidenced a decreased association. The contradiction in our study can be justified by factors related to the population, which includes the genetic backgrounds of different ethnic groups. Our genetic makeup does not belong to Chinese races in Asia. Our two ethnic groups in this study are more related to the Indian genetic model, whereas those from KPK are related to Jewish and Greek ancestry.^22^

ANXAA1 is calcium dependent phospholipid binding proteins that are involved in cancer and metastasis. In different cancers ANXA1 expressions are either up regulated or down regulated. *miR-196a* can act as a tumor suppressor, as well as an oncogene by directly targeting the *HOX* genes and *ANXA1* genes, which in turn by a negative feedback loop affect the *miR-196a* expression levels in breast cancer.^23^

We tried to establish the relationship of *miR-196a* polymorphism and mRNA expression of its target gene *ANXA1* with clinicopathological features, receptor status and genotype in our population. Changes in the expression levels of ANXA1 were noted in pathological type, and with triple negative receptor status. This means that it’s low or absent expression levels are associated with poor prognosis. Wang *et al*. observed low expressions in 56.3% of cases and more negative expression in advance disease stage.^24^ Cao *et al*. noted down regulation of ANXA1 in 79% of BC cases.^5^ While Deng *et al*. observed 80% down regulation.^25^

### Limitations of the study

In depth analysis of the mechanism of action of *miR-196a* and its various genotypes on its target genes warrants further study. The strength of this study is that, for the first time, we have assessed the genotypes of *miR-196a* in the Pakistani population and its relationship to cancer susceptibility along with its target gene ANXA1.

## CONCLUSIONS

Our study showed that the *miR-196a* (rs11614913) C/T polymorphism produced a decreased susceptibility to breast cancer different ethnicities of Pakistani population. ANXA1 expression is low in breast cancer and negative expression is associated with the homozygous CC genotype and the C/T variant of *miR-196a* rs11614913.

### Authors’ Contribution:

**AR** did concept, design and manuscript writing, is responsible for integrity of research.

**MA** conducted experimental work, data analysis.

**AKN** did review and final approval of manuscript.
